# Effect of Continuous Multi-Walled Carbon Nanotubes on Thermal and Mechanical Properties of Flexible Composite Film

**DOI:** 10.3390/nano6100182

**Published:** 2016-10-12

**Authors:** Ji Eun Cha, Seong Yun Kim, Seung Hee Lee

**Affiliations:** 1 Multifunctional Structural Composite Research Center, Institute of Advanced Composite Materials, Korea Institute of Science and Technology (KIST), 92 Chudong-ro, Bongdong-eup, Wanju-gun, Jeonbuk 55324, Korea; t15002@kist.re.kr; 2 Department of Bio, Information and Nano (BIN) Fusion Technology, Chonbuk National University, 664-14 Dukjin-gu, Jeonju-si, Jeonbuk 54896, Korea

**Keywords:** carbon nanotube, composite, film, thermal conductivity, mechanical strength

## Abstract

To investigate the effect of continuous multi-walled carbon nanotubes (MWCNTs) on the thermal and mechanical properties of composites, we propose a fabrication method for a buckypaper-filled flexible composite film prepared by a two-step process involving buckypaper fabrication using vacuum filtration of MWCNTs, and composite film fabrication using the dipping method. The thermal conductivity and tensile strength of the composite film filled with the buckypaper exhibited improved results, respectively 76% and 275% greater than those of the individual MWCNT-filled composite film. It was confirmed that forming continuous MWCNT fillers is an important factor which determines the physical characteristics of the composite film. In light of the study findings, composite films using buckypaper as a filler and polydimethylsiloxane (PDMS) as a flexible matrix have sufficient potential to be applied as a heat-dissipating material, and as a flexible film with high thermal conductivity and excellent mechanical properties.

## 1. Introduction

Since the first discovery of carbon nanotubes (CNTs) nearly two decades ago by Iijima [1], their excellent electrical, thermal and mechanical properties have been widely reported [1,2,3,4,5]. In particular, CNTs have been demonstrated to be nanomaterials with high thermal conductivity of 1950–5000 W/m·K at room temperature [1,6]. However, when typical methods of fabricating composites filled with CNTs are used, it has proven difficult to obtain the expected physical properties because of the non-uniform dispersion of the CNTs. This poor dispersion results from CNT agglomeration due to strong van der Waals forces and the extra-large surface areas of the CNTs. As methods to overcome the problem, various studies have investigated chemically modifying the CNT interface [7,8], or using continuous CNT fibers (yarns) [9,10,11] or CNT films (mats, known as buckypapers) [1,12,13].

Buckypaper is known for its outstanding electrical conductivity of 10^4^–10^7^ S/m [14,15], thermal conductivity of 83 W/m·K [16] and mechanical modulus of 0.6–4.2 GPa [17]. Furthermore, the material is advantageous for controlling both the dispersion and high loading of CNT fillers within composites [12,18]. The various buckypaper production methods can be divided into solution strategies, such as vacuum filtration [18], the spray method [19], the casting method [20], and the printing method [21], and dry strategies that do not use a solvent, such as the floating catalyst chemical vapor deposition process [22] and drying spinning [23]. Buckypaper can be used in various fields, such as filtration and distillation membranes [24], actuators [25] and supercapacitors [26], as well as thermally and electrically conductive materials [27].

**Figure 1:**
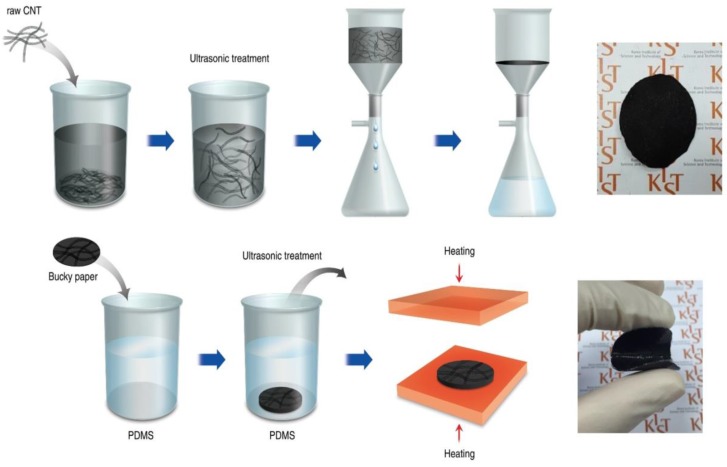
Schematic of the proposed two-step process involving buckypaper fabrication using vacuum filtration of MWCNTs and composite film fabrication using the dipping method.

The motivation of this study is to investigate the effect of incorporating individual MWCNTs or continuous MWCNT buckypaper on the thermal and mechanical properties of composite films. As shown in Figure 1, a two-step process involving buckypaper preparation via low-cost vacuum filtration and composite film fabrication based on the dipping method is proposed. Composite films filled with the individual MWCNTs, and with infiltrated MWCNT buckypaper, were fabricated by the proposed process. We evaluated the thermal and mechanical properties of the composite films and investigated the structure-property relationship resulting from forming the continuous MWCNTs.

## 2. Methods

### 2.1. Materials

**Figure 2:**
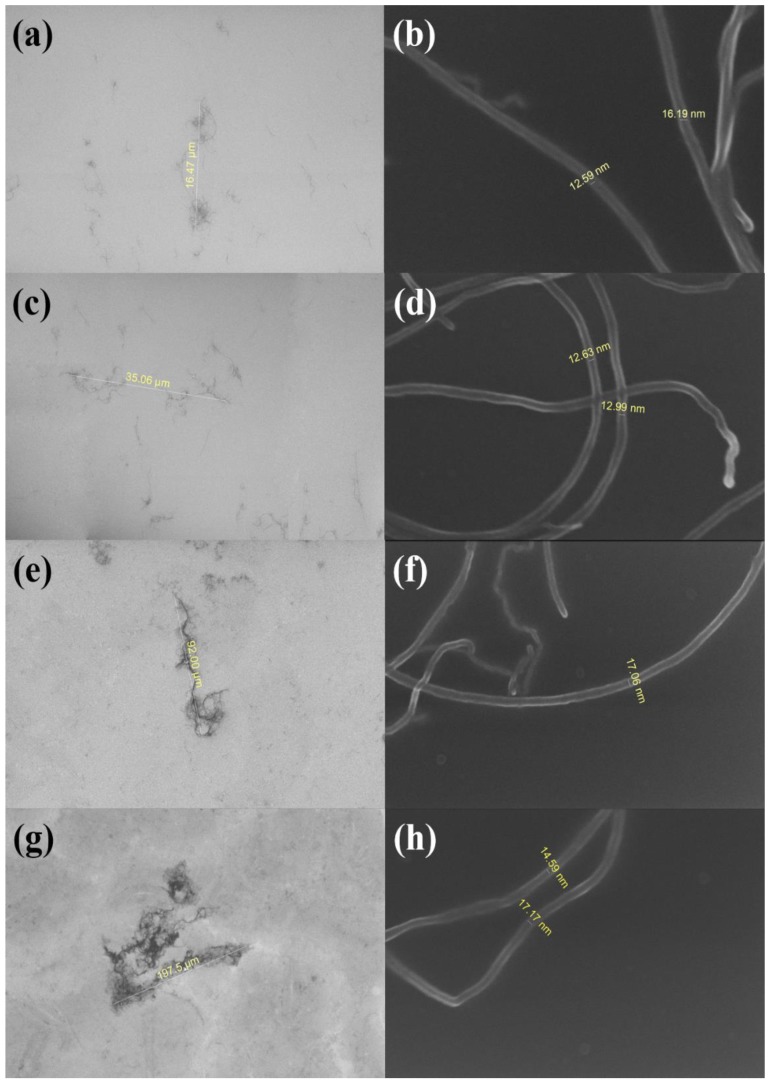
FE-SEM images of (**a**) length and (**b**) diameter of CM-130; (**c**) length and (**d**) diameter of CM-150; (**e**) length and (**f**) diameter of CM-250; and (**g**) length and (**h**) diameter of CM-280 MWCNTs.

Four types of MWCNTs (HANOS CM-130, CM-150, CM-250, and CM-280, Hanwha Nanotech, Seoul, Korea) were used as fillers, with diameters of 12–18 nm and lengths of 20, 40, 100 and 200 μm, respectively, as shown in field emission scanning electron microscopy (FE-SEM) images of Figure 2. The PDMS was used as a flexible matrix by mixing the liquid pre-polymer (Sylgard 184A, Dow Corning, Seoul, Korea) and a curing agent (Sylgard 184B, Dow Corning, Seoul, Korea) at a ratio of 10:1. The tensile strength and thermal conductivity values of the PDMS matrix reported in the literature were 1–9 MPa [28,29,30,31] and 0.15–0.2 W/m·K [31,32], respectively.

### 2.2. Preparation of MWCNT Buckypaper

Preparing a well-dispersed MWCNT aqueous suspension is important for obtaining excellent buckypaper structure and properties because the buckypapers are an entangled network structure of MWCNTs, fabricated by suspension filtration [18]. MWCNTs were dried at 100 °C for 12 h to remove moisture because it affects the dispersion of the MWCNTs. After loading the MWCNTs into a 50 mL dispersion medium according to various MWCNT types and contents, the MWCNTs were dispersed using a bath-type sonicator (JAC-3010, KODO, Hwaseong, Korea) for 20 min. Various buckypapers were produced by the vacuum filtration as shown in Figure 1 in order to investigate the effect of the MWCNT lengths, the MWCNT contents in suspension, sonication time, types of dispersion medium and types of filter on the resulting buckypaper structure, as listed in Table 1. After the filtration, buckypaper was peeled from the filter and dried overnight in a vacuum oven at 120 °C.

### 2.3. Fabrication of Composite Film

Since residual moisture interferes with silicon resin impregnation, the as-received MWCNTs and fabricated buckypaper were dried at 120 °C for 12 h in a vacuum oven. To fabricate fully impregnated PDMS composite films, the dried MWCNTs or buckypaper was immersed in the liquid phase silicon matrix mixed the liquid pre-polymer and curing agent at a ratio of 10:1 and then sonicated for 1 h using a bath-type sonicator (JAC-3010, KODO, Hwaseong, Korea). After the sonication, the PDMS composites filled with the fully dispersed MWCNTs or buckypaper were heated by a heating press (Dae Heung Science Co., Incheon, Korea) at 130 °C and 800 psi. To prepare a control sample, PDMS film without any fillers was cured under the same processing procedure and conditions. Thickness of PDMS, MWCNTs filled composite and the optimized buckypaper, sample (l) in Table 1, filled composite film was 150 μm.

### 2.4. Characterization

#### 2.4.1. Morphology

The morphology of the MWCNTs, buckypapers and fabricated composite films were observed using a field emission scanning electron microscope (Nova NanoSEM 450, FEI Corp., Hillsboro, OR, USA). Prepared samples were surface-coated with platinum for 120 s in a vacuum using a sputter coating machine (Ion Sputter E-1030, Hitachi High Technologies, Tokyo, Japan). The coated samples were observed with a voltage of 10 kV applied under nitrogen vacuum conditions.

#### 2.4.2. Fourier Transform Infrared (FT-IR) Measurements

The changes in the surface functional groups on the surface of the MWCNTs and buckypaper were analyzed by a FT-IR spectroscope (Nicolet iS10, Thermo Fisher Scientific Inc., Waltham, MA, USA). Specimens were ground with dried spectroscopic grade KBr powder and the mixture was compressed into pellets for FT-IR measurements. The FT-IR spectra were obtained in the range of 500–4000 cm^−1^ at a resolution of 16 cm^−1^.

#### 2.4.3. X-ray photoelectron spectroscopy (XPS) Measurements

XPS (K-Alpha, Thermo Fisher Scientific Inc., Waltham, MA, USA) was performed to determine the chemical compositions of the MWCNTs and buckypaper surfaces. The instrument was equipped with a monochromatic Al kα source and the measurements were performed under a pressure of $5\times 10^{-8}$ Torr.

#### 2.4.4. Raman Measurements

Raman spectroscopic analysis was performed to investigate the surface defects on the as-received MWCNTs and buckypaper using a Raman spectrometer (Renishaw Invia Reflex Raman microscope, Hoffman Estates, IL, USA) equipped with a 50× objective, and the excitation wavelength of 514.5 nm (Ar ion laser).

#### 2.4.5. Thermal Conductivity

The thermal conductivity of the composites was measured under normal temperature and pressure conditions using a conductivity-measuring instrument (TPS 2500 S, Hot Disk ab, Gothenburg, Sweden), according to the ISO 22007-2 standard. The sensor used in this study consisted of a double spiral of thin nickel wire and served as a continuous plane heat source. The sensor induced a temperature rise (ΔT) by supplying a constant amount of power (P) and, at the same time, measured the temperature change using a variation in sensor resistance. The thermal conductivity of the samples was determined using the Fourier equation for heat conduction based on the supplied power and induced temperature change.

#### 2.4.6. Heat Dissipation Property

The transient temperature response of the fabricated composite films was evaluated using an infrared camera (FLIR T420, Wilsonville, OR, USA). The composite film specimens, at a constant temperature of 100 °C, were placed on a metal plate at room temperature. Thermal images of the specimens were recorded during 20 s.

#### 2.4.7. Mechanical Property

The tensile strength of the fabricated films (3 mm × 30 mm) were tested with a Universal testing machine (UTM, UTM 5567A, Instron, Norwood, IL, USA), which was operated at a tension speed of 0.3 mm/min at room temperature.

## 3. Results and Discussion

### 3.1. Effect of Processing Conditions on Buckypaper Structure

**Figure 3:**
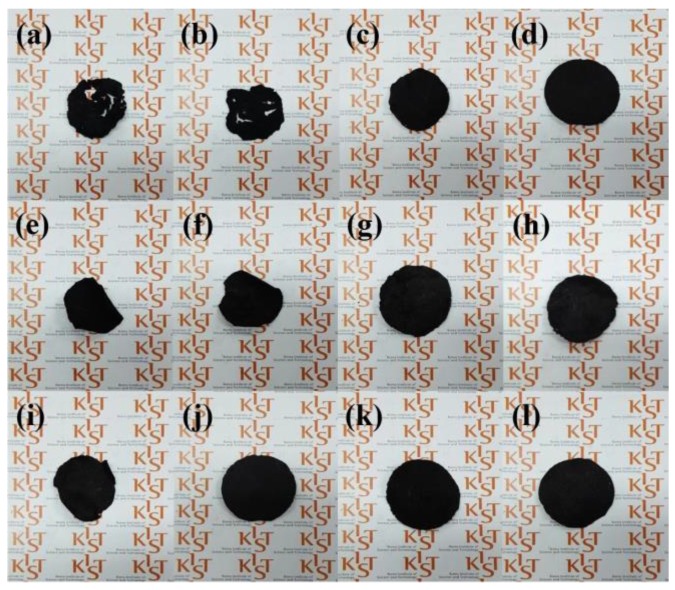
Photo images of buckypapers with fabrication conditions. The sample names were listed in Table 1.

The buckypaper fabrication conditions and photo images are listed in Table 1 and shown in Figure 3, respectively. It was confirmed in Figure 3a–d that when longer MWCNTs were used, more effective contact between the MWCNTs occurred, resulting in closed-packed buckypaper [12]. When comparing the tensile strength of the fabricated buckypapers listed in Table 1, the optimum condition for fabrication of the buckypapers was 50-mg-long- MWCNTs in 50 mL DMF with 20 min sonication using the PTFE filter. DMF is known as an effective dispersion medium for making an agglomerate-free uniform MWCNT dispersion because of good interactions between the MWCNTs and DMF in the suspension phase [33]. During suspension filtration under the optimum condition, uniformly sequenced fibrous MWCNTs formed by van der Waals forces between the tubes were identified [34].

**Figure 4:**
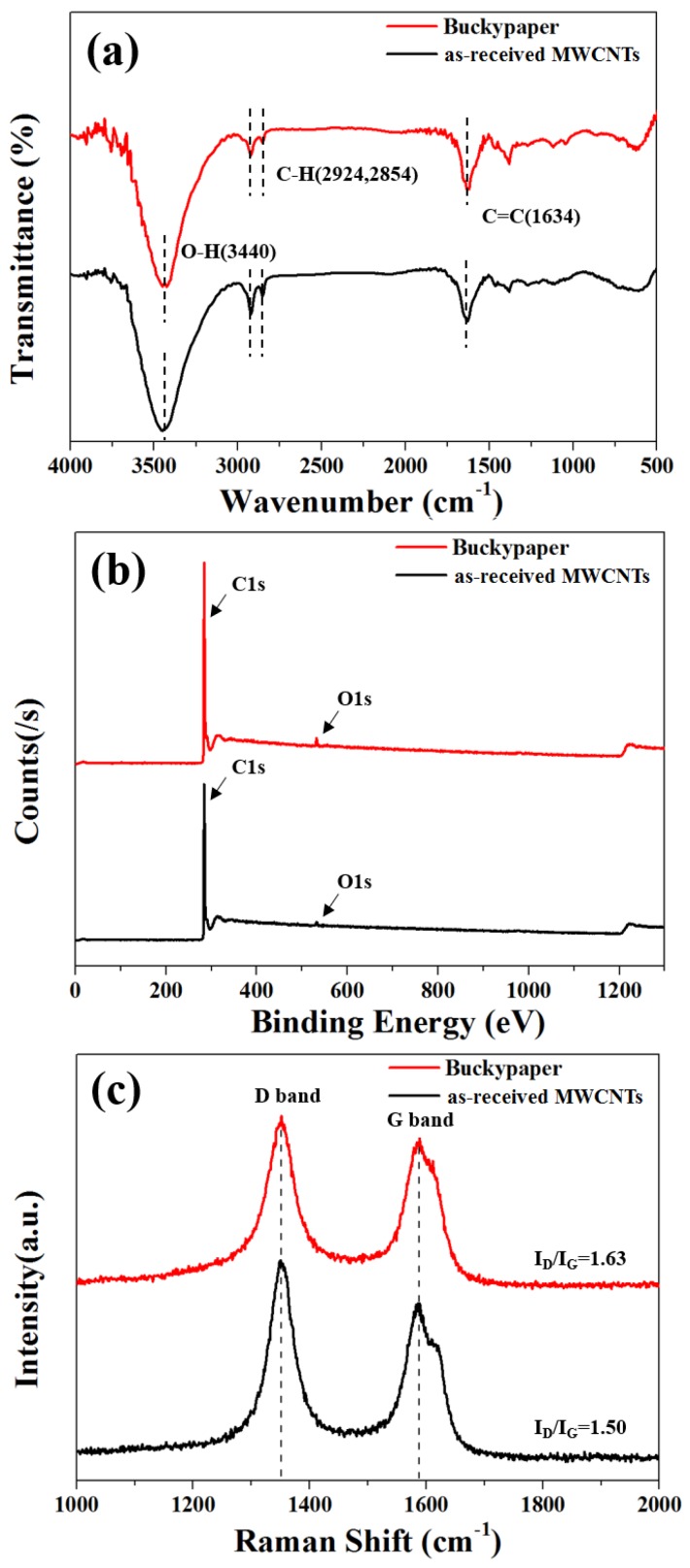
(**a**) FT-IR spectra; (**b**) XPS survey-scan spectra; and (**c**) Raman spectra of the as-received MWCNTs and buckypaper fabricated under the optimized condition.

The chemical surface properties of the as-received MWCNTs and the fabricated buckypaper are shown in Figure 4. As shown in the FT-IR spectra of the as-received MWCNTs and the buckypaper of Figure 4a, similar peaks at 3440 cm^−1^ (O–H), 2924 and 2854 cm^−1^ (C–H) and 1634 cm^−1^ (C=C) were observed. The pristine MWCNTs and buckypaper fabricated under the optimum condition using DMF solvent had a similar atomic percentage of carbon and oxygen, as confirmed in the XPS spectra of Figure 4b and Table 2. In particular, the N1s peak which can be found by reacting with the DMF solvent was not observed in the XPS survey-scan spectra of the buckypaper [35]. In the Raman spectra shown in Figure 4c, a similar intensity ratio of I_D_/I_G_ was observed for both the as-received MWCNTs and the buckypaper. The I_D_/I_G_ ratio indicates the defect level of the carbon materials because the D-band is a disorder-induced feature that arises from a double-resonance Raman scattering process due to non-zero-center phonon modes, which are typically attributed to the presence of amorphous or disordered carbon atoms, and the G band results from in-plane tangential stretching of the carbon-carbon bonds in the graphene sheets [36]. Therefore, there were no apparent structural or chemical changes during the buckypaper fabrication using DMF solvent.

### 3.2. Properties of Composite Films

**Figure 5:**
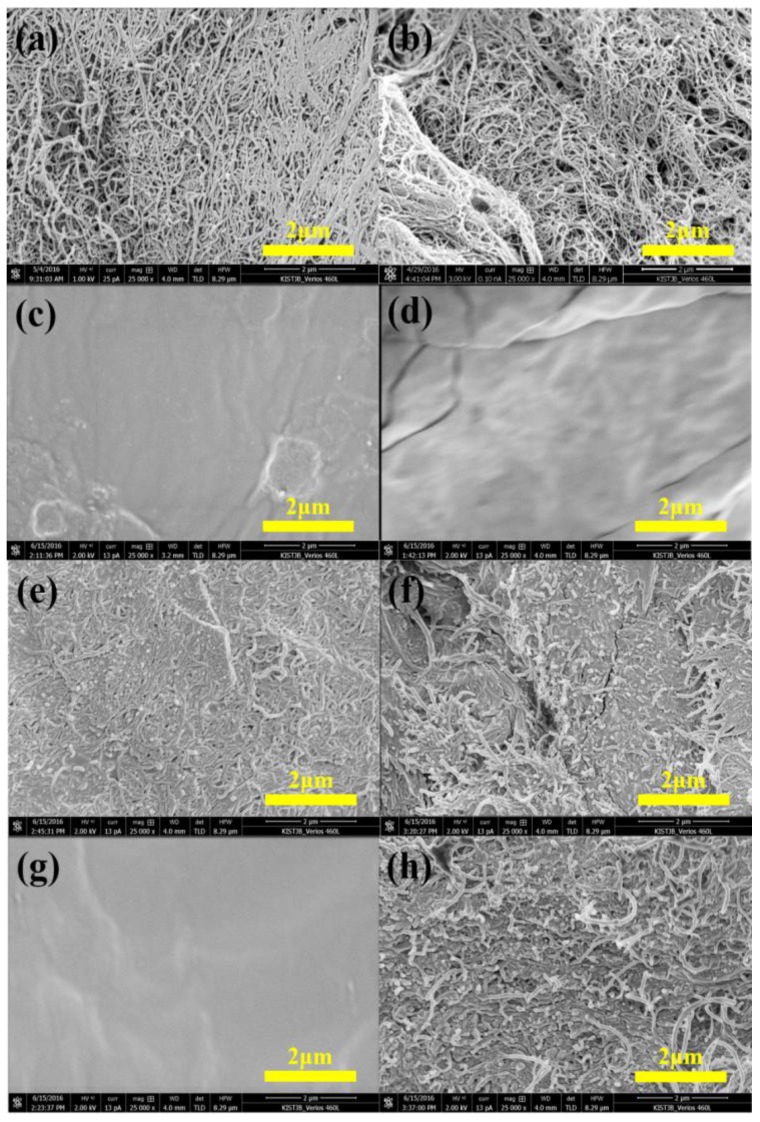
FE-SEM images of (**a**) surface and (**b**) fracture of the fabricated buckypaper; (**c**) surface and (**d**) fracture of pure PDMS film; (**e**) surface and (**f**) fracture of individual MWCNT-filled composite film; and (**g**) surface and (**h**) fracture of buckypaper-filled composite film.

The fabrication of the PDMS composite film was based on the proposed two-step process including buckypaper vacuum filtration and composite film fabrication. As a result, optimization of the composite film fabrication as well as the buckypaper vacuum filtration was required. It was possible to control the thickness of the PDMS matrix coated on the outside of the filled buckypaper by adjusting the compression pressure during the final step of the proposed processing. A photo image and FE-SEM images of the composite film filled with the buckypaper are shown in Figure 1 and Figure 5, respectively. It was verified that the PDMS matrix was fully impregnated into the buckypaper network, and the flexible PDMS composite film filled with the buckypaper was fabricated without any pores.

**Figure 6:**
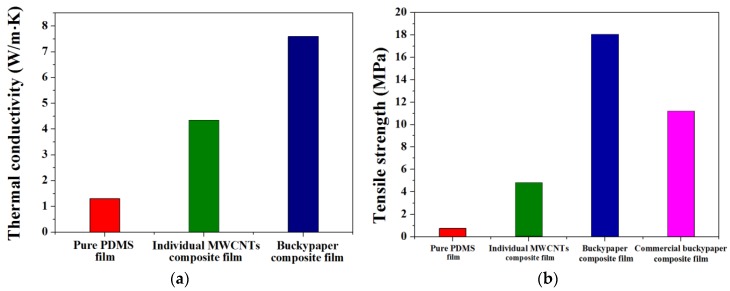
(**a**) Thermal conductivity and (**b**) tensile strength of pure PDMS film, individual MWCNT-filled composite film, buckypaper-filled composite film and commercial buckypaper-filled composite film.

**Figure 7:**
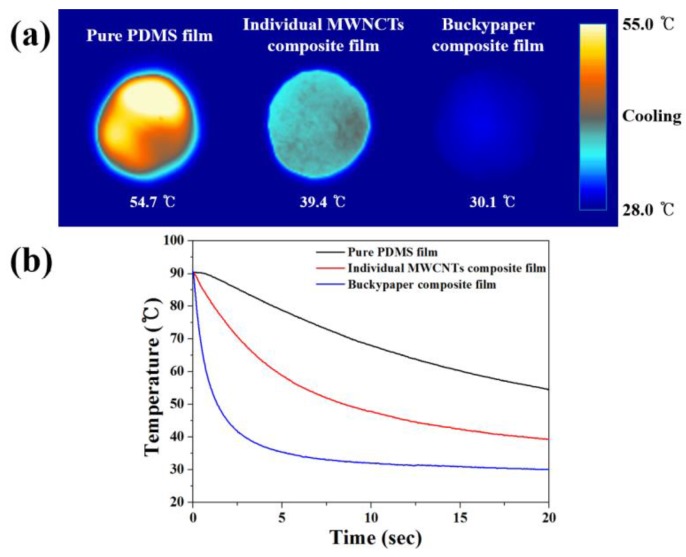
(**a**) Infrared camera images and (**b**) temperature-time profiles of the films during cooling for transient temperature responses.

Figure 6 shows the thermal and mechanical properties of the PDMS composite films. Considering the high thermal conductivity of the MWCNTs, the results for the composite films were lower than expected. The disappointing results can be explained by the fact that phonon transfer in a polymer composite filled with CNTs is impeded by the interfacial thermal resistance between the CNTs and polymer matrix, and the contact resistance between CNTs [37,38,39,40]. The in-plane thermal conductivity of the composite film filled with the buckypaper reached up to 7.6 W/m·K, a 484% and 76% increase compared to that of the pure PDMS film and individual MWCNT-filled composite film, respectively. In the buckypaper-filled composite film, the tube-tube overlap of MWCNTs composing the buckypaper, which are arranged in the horizontal direction, leads to excellent phonon transfer and high thermal conductivity [1]. The transient temperature response of the fabricated composite films is exhibited in Figure 7. The temperature decrease during cooling was considerably faster in the composite film with the highest thermal conductivity compared with the other films, indicating that the high thermal conductivity provides a high heat transfer rate. Moreover, the temperature change trend was in good agreement with the thermal conductivity trend, implying that forming the continuous MWCNTs resulted in both the high thermal conductivity and heat dissipation property of the buckypaper-filled composite film.

The tensile strengths of the composite films are shown in Figure 6b. The tensile strengths of the composite films filled with the buckypaper and as-received MWCNTs were improved by 2268% and 531% compared to that of the pure PDMS film. The buckypaper inside the composite film was able to form a broad molecular-level interaction due to the continuous and large surface area of the tube-tube overlapped MWCNTs [12]. The simultaneous enhancement of the thermal and mechanical properties is an important advantage of the composite film filled with the buckypaper. The tensile strength of the composite film filled with a commercially available buckypaper (CNP40, Nanolab, Waltham, MA, USA) fabricated under the same processing condition was also investigated. The result was measured to be 11.2 MPa and the value was as low as 6.8 MPa, indicating that the fabrication method and conditions proposed in this study for the buckypaper-filled composite film were optimized.

## 4. Conclusions

We proposed a fabrication method for buckypaper-filled flexible composite film with excellent thermal and mechanical properties which was prepared by a two-step process involving buckypaper fabrication using vacuum filtration of MWCNTs and composite film fabrication using the dipping method. The effect of incorporating individual MWCNTs and continuous MWCNT buckypaper on the thermal and mechanical properties of the composite films was investigated. The surface chemical and structural properties of the MWCNTs were not altered during the vacuum filtration processing and the flexible PDMS composite films filled with the buckypaper or as-received MWCNTs were fabricated without any pores. The thermal and tensile properties of the composite film filled with the continuous buckypaper were superior to those of the composite film filled with the individual MWCNTs due to the formation of phonon transport pathways and molecular-level interactions between MWCNTs within the buckypaper. The thermal conductivity and tensile strength of the buckypaper-filled composite film were improved by 76% and 275%, respectively, by forming the continuous MWCNT buckypaper.

## Figures and Tables

**Table 1:** Buckypaper fabrication conditions, thickness and tensile strength of the fabricated buckypapers.

Sample	Types of MWCNT	MWCNT Content (mg/50 mL)	Sonication Time (min)	Dispersion Medium	Types of Filter	Thickness (μm)	Tensile Strength (MPa)
(a)	CM-130	50	20	Ethanol	Nylon	220	-
(b)	CM-150	50	20	Ethanol	Nylon	100	-
(c)	CM-250	50	20	Ethanol	Nylon	150	0.68
(d)	CM-280	50	20	Ethanol	Nylon	150	1.98
(e)	CM-280	10	20	Ethanol	Nylon	100	-
(f)	CM-280	30	20	Ethanol	Nylon	130	1.96
(g)	CM-280	70	20	Ethanol	Nylon	200	1.20
(h)	CM-280	90	20	Ethanol	Nylon	240	0.74
(i)	CM-280	50	10	Ethanol	Nylon	170	0.75
(j)	CM-280	50	30	Ethanol	Nylon	160	1.13
(k)	CM-280	50	20	DMF ^1^	Nylon	150	2.08
(l)	CM-280	50	20	DMF	PTFE ^2^	130	3.70

^1^ Dimethylformamide; ^2^ Polytetrafluoroethylene.

**Table 2:** XPS analysis results of the as-received MWCNTs and buckypaper fabricated under the optimized conditions.

	Element	Peak Position (eV)	Atomic Concentration (%)
As-received MWCNTs	O1s	532.36	1.6
	C1s	284.37	98.4
Buckypaper fabricated under the optimized conditions	O1s	533.21	1.69
	C1s	285.31	98.31

## Abbreviations

The following abbreviations are used in this manuscript:

MWCNTs	multi-walled carbon nanotubes
CNTs	carbon nanotubes
PDMS	Polydimethylsiloxane
DMF	Dimethylformamide
PTFE	Polytetrafluoroethylene
FE-SEM	field emission scanning electron microscopy
FT-IR	Fourier transform infrared
XPS	X-ray photoelectron spectroscopy
